# Can GPR4 Be a Potential Therapeutic Target for COVID-19?

**DOI:** 10.3389/fmed.2020.626796

**Published:** 2021-01-21

**Authors:** Li V. Yang, Karen A. Oppelt, Mary Jane Thomassen, Mona A. Marie, Shayan Nik Akhtar, Justin D. McCallen

**Affiliations:** ^1^Department of Internal Medicine, Brody School of Medicine, East Carolina University, Greenville, NC, United States; ^2^Department of Anatomy and Cell Biology, Brody School of Medicine, East Carolina University, Greenville, NC, United States; ^3^Department of Comparative Medicine, Brody School of Medicine, East Carolina University, Greenville, NC, United States

**Keywords:** COVID-19, GPR4, inflammation, endothelial cell, vascular permeability, thromboembolism

## Abstract

Coronavirus disease 19 (COVID-19), caused by severe acute respiratory syndrome coronavirus 2 (SARS-CoV-2), first emerged in late 2019 and has since rapidly become a global pandemic. SARS-CoV-2 infection causes damages to the lung and other organs. The clinical manifestations of COVID-19 range widely from asymptomatic infection, mild respiratory illness to severe pneumonia with respiratory failure and death. Autopsy studies demonstrate that diffuse alveolar damage, inflammatory cell infiltration, edema, proteinaceous exudates, and vascular thromboembolism in the lung as well as extrapulmonary injuries in other organs represent key pathological findings. Herein, we hypothesize that GPR4 plays an integral role in COVID-19 pathophysiology and is a potential therapeutic target for the treatment of COVID-19. GPR4 is a pro-inflammatory G protein-coupled receptor (GPCR) highly expressed in vascular endothelial cells and serves as a “gatekeeper” to regulate endothelium-blood cell interaction and leukocyte infiltration. GPR4 also regulates vascular permeability and tissue edema under inflammatory conditions. Therefore, we hypothesize that GPR4 antagonism can potentially be exploited to mitigate the hyper-inflammatory response, vessel hyper-permeability, pulmonary edema, exudate formation, vascular thromboembolism and tissue injury associated with COVID-19.

## Introduction

COVID-19 first emerged in Wuhan, China in December 2019 and has rapidly become a global pandemic with confirmed cases in more than 200 countries and regions. By November 5, 2020, nearly 48 million confirmed cases and over 1.2 million deaths have been reported around the world (1). The pandemic is continuing to spread and more confirmed cases and COVID-related deaths are reported every day. In addition to the staggering number of human casualties and as a global effort to stop the pandemic, social distancing, stay-at-home orders, and closure of schools and businesses have caused enormous societal burdens and economic losses. Development of effective vaccines and therapeutics is critical to curb the pandemic and save the lives of patients afflicted by COVID-19.

For COVID-19 patients, disease severities span from asymptomatic infection, mild respiratory illness to severe pneumonia with respiratory failure and death (2–4). In a study of 44,415 cases in China, 81% of patients had mild symptoms, 14% had severe symptoms and 5% had critical disease manifestations (5). The worldwide mortality rate is approximately 2.5% among the confirmed cases (1,221,781/47,930,397 as of November 5, 2020) (1), with a higher mortality rate in elderly patients and those with underlying conditions such as hypertension, diabetes, and cardiovascular disease.

The pathophysiology of COVID-19 is not completely understood. SARS-CoV-2 infects a wide range of cells, including type II pneumocytes, vascular endothelial cells, pericytes, macrophages, T cells, cardiomyocytes, enterocytes, kidney epithelial cells and podocytes, all of which express the SARS-CoV-2 receptor ACE2 (angiotensin converting enzyme 2) (4, 6). Airway epithelial cells infected by SARS-CoV-2 trigger an inflammatory response, with production of increased levels of cytokines and chemokines that stimulate the infiltration of neutrophils, monocytes, and lymphocytes into the lung and other target organs (4, 6). Autopsy studies of patients succumbing to COVID-19 have revealed some key pathological findings, such as diffuse alveolar damage, inflammatory cell infiltration, pulmonary edema, proteinaceous exudates, and vascular thromboembolism in the lung, which potentially contribute to disease severity, acute respiratory distress syndrome (ARDS) and respiratory failure in the patients (7–9). In addition to lung injuries, COVID-19 complications also include impaired function of the liver, kidney, heart, brain, and coagulation system (4, 6, 10).

## Hypothesis

We propose that GPR4 is involved in COVID-19 pathophysiology and can be exploited as a potential therapeutic target for COVID-19. GPR4 is a pro-inflammatory GPCR that regulates endothelial cell adhesion, leukocyte infiltration, blood vessel permeability, and angiogenesis (11–20). GPR4 is expressed in various tissues, with high expression in the lung, heart, and kidney (21, 22). The cell types predominantly expressing GPR4 are vascular endothelial cells and GPR4 gene expression is also found in other cell types such as neurons, kidney epithelial cells, osteoblasts, and chondrocytes (12, 14, 23–26). Biochemically, GPR4 can be activated by extracellular protons (acidosis), with acidotic conditions commonly existing in inflamed tissues due to hypoxia and glycolytic cell metabolism (27, 28). Importantly, genetic and pharmacological inhibition of GPR4 alleviates inflammatory responses, reduces leukocyte infiltration, and decreases tissue edema in several animal models of inflammatory disorders including arthritis, inflammatory bowel disease, chronic obstructive pulmonary disease (COPD), and ischemic diseases (14, 15, 17–20, 29). Many of the GPR4-regulated inflammatory processes described above share cardinal pathological features observed in COVID-19 patients (7–9). Therefore, we hypothesize that GPR4 plays a role in COVID-19 pathophysiology and GPR4 antagonism is a potential therapeutic approach to mitigate COVID-19 complications.

## Evaluation of the Hypothesis

### Does GPR4 Play a Role in the Pathophysiology of COVID-19?

#### Pathophysiology of Inflammatory Responses in COVID-19

COVID-19 is caused by the infection of SARS-CoV-2, a novel β-coronavirus sharing ~88% and ~80% sequence homology with the bat derived SARS-like coronaviruses and SARS-CoV, respectively (30). Similar to SARS-CoV, the spike glycoprotein (S protein) of SARS-CoV-2 binds to cell surface ACE2 receptors to gain entry into cells (4, 6). In the early stage of disease, SARS-CoV-2 targets the respiratory system, infecting nasal and bronchial epithelial cells and lung pneumocytes and stimulating inflammatory responses in these cells. Consequently, the infected cells produce increased levels of cytokines and chemokines, such as interleukin-6 (IL-6), IL-1, TNF-α, CXCL10 (IP-10), CCL2 (MCP-1), and CCL3 (MIP-1α). The cytokines and chemokines, in turn, induce massive infiltration of leukocytes into the lung. The accumulation of neutrophils, monocytes, and macrophages in the lung further increases the production of cytokines and chemokines, generating a vicious cycle of inflammation. Excessive production of cytokines can lead to “cytokine storm,” acute respiratory distress syndrome (ARDS), tissue injury, multi-organ failure, and death in critically ill COVID-19 patients (4, 6).

Besides airway epithelial cells, SARS-CoV-2 also infects many other types of cells expressing the ACE2 receptor, such as vascular endothelial cells and pericytes (4, 6, 10). SARS-CoV-2 infection of lung microvascular endothelial cells aggravates the inflammatory response. Endothelial cells function as a physiological interface to interact with leukocytes and platelets. Pulmonary endothelial and epithelial barriers are critical for the regulation of gas exchange and immune cell recruitment in the lung. SARS-CoV-2 infection of alveolar epithelial cells and pulmonary endothelial cells can cause cell death and stimulate inflammatory responses. Consequently, disruption of pulmonary endothelial and epithelial barriers leads to excessive leukocyte infiltration into the lung, plasma fluid flooding into interstitial and alveolar spaces (i.e., permeability edema), shortness of breath, hypoxemia, pneumonia, and ARDS in COVID-19 patients.

In addition to the hyper-inflammation and damage to pulmonary epithelial and endothelial barriers, vascular thromboembolism in the lung and other organs is also a common complication observed in severe COVID-19 patients that is associated with fatal outcomes. In a study of 184 patients admitted to the intensive care unit (ICU), thrombotic complications were observed in 31% of the patients (31). Autopsy studies of the lungs from seven patients who died from COVID-19 showed widespread thrombosis in pulmonary vessels and microthrombi in alveolar capillaries (32). Increased new blood vessel growth (angiogenesis) was also observed in the lungs of these patients (32). Vascular thromboembolism is closely associated with mortality in COVID-19 patients. Predisposition to thromboembolism is believed to be due to excessive inflammation and coagulopathy in COVID-19 patients (31, 32).

Extrapulmonary complications of COVID-19 have been observed in multiple organ systems, such as the cardiovascular, hematological, gastrointestinal, renal, endocrine, dermatological and neurological systems (4, 6, 10). While COVID-19 is caused by SARS-CoV-2 infection, the host inflammatory response also plays critical roles in the pathophysiology of the disease. Overall, SARS-CoV-2 infection-induced direct cytotoxicity, hyper-inflammation, endothelial cell dysfunction, thromboembolism, cytokine-release syndrome, and dysregulation of the renin-angiotensin-aldosterone system (RAAS) are considered as the major mechanisms responsible for systemic COVID-19 complications in multiple organ systems (4, 6, 10).

#### How Is GPR4 Potentially Involved in the Pathophysiology of COVID-19?

As described above, hyper-inflammatory responses in patients with increased levels of cytokines, chemokines, leukocyte infiltration, vascular permeability, tissue edema, endothelialitis, and thromboembolism represent some key pathophysiological features in COVID-19 (4, 6). Herein, we evaluate the potential involvement of GPR4 in the pathophysiology of COVID-19.

GPR4 is highly expressed in vascular endothelial cells and has emerged as a key regulator of inflammatory responses (11–20, 27). As a proton-sensing GPCR, GPR4 can be activated by acidosis which is a microenvironment hallmark of numerous pathological conditions such as inflammation, ischemia, and tumors (27, 28). Activation of GPR4 by acidosis stimulates the expression of inflammatory chemokines, cytokines, adhesion molecules and the NF-κB pathway in endothelial cells, increases endothelium-leukocyte adhesion, and facilitates leukocyte infiltration (11–16, 20). Moreover, activation of GPR4 by acidosis promotes the endoplasmic reticulum (ER) stress response and apoptosis of endothelial cells (12, 13, 33).

Multiple lines of evidence support a pro-inflammatory role of GPR4 in various pathological conditions (11–20, 27, 29). Using GPR4 knockout (KO) mice, studies demonstrated that GPR4 deletion reduces inflammation in mouse colitis models (14, 19). In the dextran sulfate sodium (DSS)-induced acute colitis mouse model, GPR4 deletion ameliorates intestinal inflammation (14). The indicators of disease severity, such as body weight loss, mesenteric lymph node expansion, colon shortening, fecal diarrhea score, and intestinal histopathology, are alleviated in the GPR4 KO mice compared to wild-type mice. GPR4 deletion reduces the expression of endothelial adhesion molecules E-selectin and VCAM-1 in the colon of the DSS-induced colitis mice (14). Another study also demonstrated that GPR4 deletion alleviates intestinal inflammation in the DSS-induced colitis and the IL10-/- spontaneous colitis mouse models (19). Interestingly, GPR4 mRNA is over-expressed by approximately 5-fold in the inflamed intestinal lesions of inflammatory bowel disease (IBD) patients when compared to normal intestinal tissues (14). Furthermore, in the tourniquet cuff-induced hindlimb ischemia-reperfusion mouse model, GPR4 deletion reduces inflammatory response, leukocyte infiltration, vascular permeability, tissue edema and proteinaceous exudate formation in the limb tissue (20).

**Figure 1:**
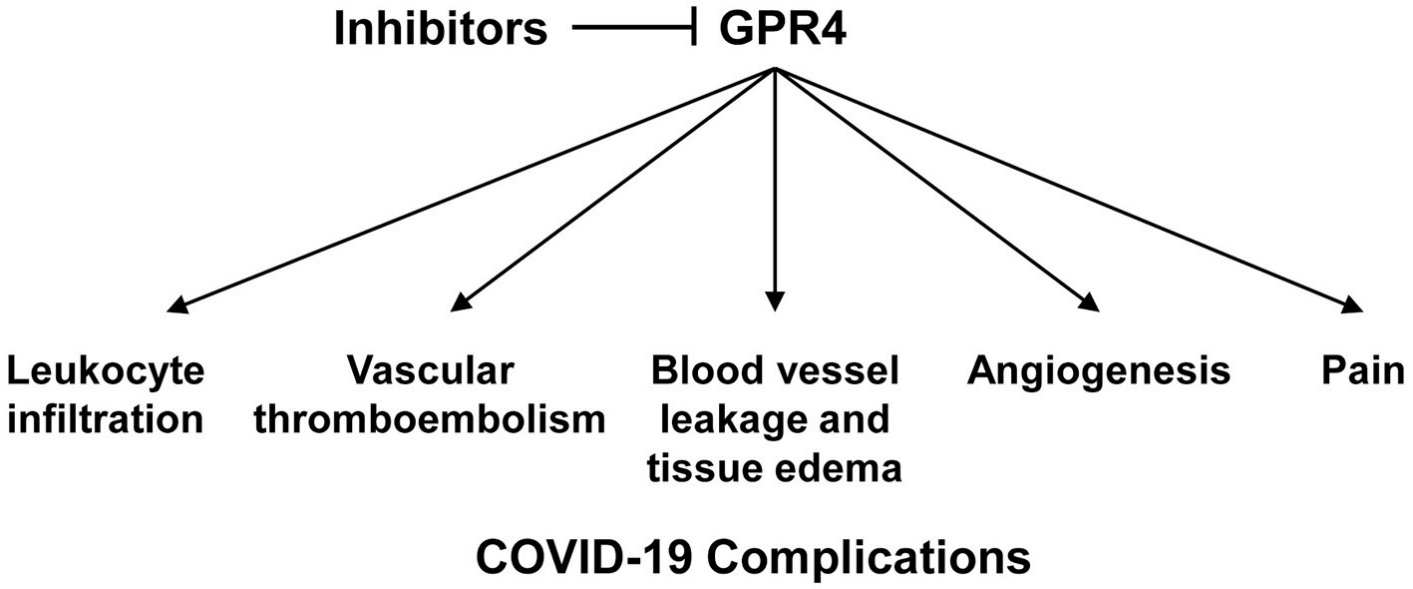
A hypothetical model to depict the roles of GPR4 in COVID-19 pathophysiology and the inhibition of GPR4 as a potential approach to mitigate COVID-19 complications.

Based on its biological functions, GPR4 can potentially regulate multiple aspects of COVID-19 pathophysiology (Figure 1). SARS-CoV-2 infection of lung epithelial cells and endothelial cells induces inflammatory responses in these cells with increased expression of cytokines and chemokines (4, 6). As the disease severity of COVID-19 progresses, alveolar epithelial and endothelial barriers become disrupted, oxygen/carbon dioxide exchange is impaired, and the lung tissues become hypoxic. The pH of inflammatory and hypoxic tissues is acidic due to reduced oxygen levels, glycolytic cell metabolism, and proton accumulation (27, 28, 34–36). In addition to acidotic pH in inflamed and hypoxic tissues, respiratory and metabolic acidosis is a common complication observed in COVID-19 patients, especially in patients with severe disease (37). Also, COVID-19 may aggravate ketoacidosis in diabetes patients and cause kidney injuries, leading to metabolic acidosis in patients (38–40). As a proton-sensing GPCR, GPR4 is optimally activated under acidic extracellular pH (6.4–6.9) and partially activated at physiological pH 7.4 (41, 42). As described above, activation of GPR4 increases the expression of inflammatory adhesion molecules, chemokines, and cytokines in vascular endothelial cells, which can in turn enhance leukocyte infiltration (11, 12, 14, 16). Increased inflammation and adhesiveness of endothelial cells can be prothrombotic and stimulate the adhesion and aggregation of platelets and leukocytes (43). Moreover, activation of GPR4 augments paracellular gap formation and permeability of endothelial cells, which can lead to fluid accumulation and edema in the tissues (17, 18, 20). All these biological functions of GPR4 are highly relevant to COVID-19 patient pathophysiology, including the hyper-inflammatory response, leukocyte infiltration, blood vessel leakage, pulmonary edema, and vascular thromboembolism (4, 6). Moreover, GPR4 gene expression is up-regulated in COVID-19 patient samples. A recent study used RNA sequencing (RNA-Seq) to identify differentially expressed genes (DEGs) in lung and colon samples from patients succumbing to COVID-19 (44). Compared to normal lung and colon samples, GPR4 mRNA levels were increased by 2.3-fold (*p* = 3.04E-06) and 3.9-fold (*p* = 0.0074), respectively, in COVID-19 patient lung and colon samples. The up-regulation of GPR4 gene expression in COVID-19 patient tissues may further aggravate the GPR4-mediated pro-inflammatory effects and contribute to COVID-19 pathophysiology.

### Can GPR4 Antagonism Provide Therapeutic Benefits for the Treatment of COVID-19?

#### Current Treatment Approaches for COVID-19

Current therapeutic modalities for COVID-19 mainly include anti-viral drugs, antibodies, and anti-inflammatory agents. A randomized clinical trial demonstrated that the treatment with the antiviral drug remdesivir accelerates the recovery of hospitalized COVID-19 patients (11 vs. 15 days, compared to placebo, *P* < 0.001) and also shows a trend of survival benefit (a mortality rate by 14 days of 7.1 vs. 11.9%, compared to placebo, *P* > 0.05) (45). However, another randomized clinical trial failed to demonstrate significant therapeutic benefits of remdesivir in patients with severe COVID-19 (46). Transfusion with convalescent plasma containing antiviral neutralizing antibodies demonstrates signs of improvement in critically ill COVID-19 patients. In 35,322 severely ill COVID-19 patients (52.3% in the ICU and 27.5% receiving mechanical ventilation) transfused with convalescent plasma on the Expanded Access Program, a decrease of 30-day mortality was observed in patients transfused within 3 days of COVID-19 diagnosis compared to 4 or more days after diagnosis (21.6 vs. 26.7%, *p* < 0.0001), suggesting a potential benefit of early intervention. Also, the mortality of the patients receiving high IgG plasma was lower than that of the patients receiving low IgG plasma (47). However, a phase II randomized trial with hospitalized, moderately ill COVID-19 patients (235 in the intervention arm and 229 in the control arm) failed to demonstrate any reduction in mortality with convalescent plasma treatment (14.5% in the intervention arm vs. 13.5% in the control arm) (48). In a phase II randomized trial involving outpatients with mild or moderate COVID-19, the SARS-CoV-2 neutralizing antibody LY-CoV555 was shown to decrease viral load and reduce the percentage of patients who had a COVID-19 related hospitalization or emergency department visit (1.6 vs. 6.3%, compared to placebo) (49). In addition to antiviral drugs, convalescent plasma and neutralizing antibodies, studies have evaluated the therapeutic effects of anti-inflammatory agents in COVID-19 because hyper-inflammatory responses (cytokine storm) are observed in some COVID-19 patients. The Recovery trial showed that dexamethasone reduces mortality rate in hospitalized COVID-19 patients within 28 days (22.9% in the dexamethasone group vs. 25.7% in the usual care group, *P* < 0.001), with a trend of more benefits in patients receiving invasive mechanical ventilation or oxygen (50). Tocilizumab, a monoclonal antibody targeting the interleukin-6 receptor (IL-6R), demonstrated clinical benefits in some COVID-19 patients by reducing the hyper-inflammatory responses; however, it was also observed that some patients were refractory to tocilizumab treatment (51, 52). This is likely because a multitude of inflammatory cytokines and molecules are involved in the hyper-inflammatory response. In a recent open-label cohort study of severe COVID-19 patients with systemic hyper-inflammation, blockade of IL-6R with sarilumab did not significantly improve overall clinical outcomes or reduce mortality compared to standard of care, but sarilumab treatment was associated with faster recovery in a subset of patients (53). Overall, current therapeutic approaches for COVID-19 patients with severe disease are not particularly effective. It is critical to fully understand the pathophysiology of COVID-19 and develop more effective therapeutics to significantly reduce COVID-19 mortality and morbidity.

#### Can GPR4 Antagonism Mitigate COVID-19 Complications?

Recently, GPR4 specific inhibitors have been developed and their biological effects have been characterized (Table 1). Derivatives of imidazo-pyridine and pyrazolopyrimidine compounds have been identified as novel GPR4 antagonists (16–18, 54). Studies have shown that GPR4 antagonists reduce inflammation in the antigen-induced arthritis rat model, the DSS-induced acute colitis mouse model, and the short-term emphysema-exacerbation COPD mouse model as well as alleviate inflammatory pain in the complete Freund's adjuvant-induced hyperalgesia rat model (15, 17, 18, 29). In the DSS-induced acute colitis model, GPR4 antagonist 13 (NE-52-QQ57) ameliorates intestinal inflammation and decreases the expression of TNF-α in the inflamed mouse colon tissues (15). In the COPD mouse model, treatment with GPR4 antagonist reduces leukocyte infiltration, inflammatory cytokine expression, mucin production, and protease expression in the lung (29). Like GPR4 genetic knockout mice, GPR4 antagonists exhibit anti-angiogenic effects and attenuate inflammatory responses, tissue edema and exudate formation (17, 18, 20). Additionally, GPR4 antagonists reduce the expression of inflammatory chemokines, cytokines, adhesion molecules, NF-κB pathway genes, and stress responsive genes, such as IL-1, IL-8, CXCL1, CXCL2, CCL2, CCL7, VCAM-1, ICAM-1, E-selectin, RELB, COX2, ATF3, and CHOP, in cultured endothelial cells (12, 13, 16). Treatment with the GPR4 antagonist NE-52-QQ57 inhibits the expression of inflammatory molecules including TNF-α, IL-1β, IL-6, iNOS, nitric oxide (NO), COX2, and PGE2 in cultured chondrocytes (24). Furthermore, studies have demonstrated that genetic knockout and pharmacological inhibition of GPR4 protect mice from ischemic injury in the myocardial infarction, renal ischemia-reperfusion, and hindlimb ischemia-reperfusion mouse models (20, 54, 55).

**Table 1:** Biological effects of GPR4 antagonists *in vitro* and *in vivo*.

**Experimental models**	**GPR4 antagonist effects**	**References**
Endothelial cell culture (*in vitro*): microarray, qRT-PCR, and Western blot analyses; endothelial cell adhesion, gap formation, and permeability assays	Inhibiting the expression of inflammatory chemokines, cytokines, adhesion molecules, COX2, NF-κB pathway genes, and ER stress genes in endothelial cells in response to acidosis; Reducing endothelial cell-leukocyte adhesion; Decreasing endothelial paracellular gap formation and permeability.	(12, 13, 16, 20)
Chondrocyte culture (*in vitro*): SW1353 chondrocyte cell line; RT-PCR, Western blot, and ELISA analyses; nitric oxide assay; NF-κB reporter assay	Inhibiting the advanced glycation end products (AGEs)-induced expression of inflammatory molecules such as TNF-α, IL-1β, IL-6, iNOS, nitric oxide (NO), COX2, and PGE2; Inhibiting the expression of matrix metalloproteinase (MMP)-3 and MMP-13; Suppressing the NF-κB pathway.	(24)
Mouse myocardial infarction model (*in vivo*): Kaplan–Meier survival analysis	Prolonging mouse survival in the myocardial infarction model with permanent left anterior descending coronary artery ligation.	(54)
Rat antigen induced arthritis model (*in vivo*): rats sensitized with methylated bovine serum albumin (mBSA)/complete Freund's adjuvant (CFA)	Reducing knee swelling, inflammatory cell infiltration, joint damage, and proteoglycan loss, comparable to the effects of dexamethasone.	(17, 18)
Rat hyperalgesia model (*in vivo*): inflammatory pain induced by CFA	Demonstrating antinociceptive effects, comparable to diclofenac, in the complete Freund's adjuvant (CFA) induced hyperalgesia rat model.	(17, 18)
Mouse angiogenesis model (*in vivo*): porous tissue chambers filled with VEGF	Inhibiting VEGF-induced angiogenesis.	(17, 18)
Mouse and rat cardiorespiratory models (*in vivo*): evaluation of the GPR4 antagonist NE 52-QQ57 on cardiorespiratory effects in rodents	Having no effects on hemodynamics, cerebral blood flow, and blood oxygen level dependent responses in anesthetized rats; Causing a small reduction in the ventilatory response to 5 and 10% CO_2_ in awake but not in anesthetized mice and rats; Having no serious adverse effects on cardiovascular and respiratory systems in rodents.	(23)
Mouse colitis model (*in vivo*): DSS-induced colitis model studied by gene expression and histopathologic analyses	Alleviating intestinal inflammation in the DSS-induced colitis mouse model; Attenuating leukocyte infiltration in the colon; Reducing mesenteric lymph node enlargement; Decreasing the expression of VCAM-1, E-selectin, and TNF-α in colon blood vessels.	(15)
Mouse hindlimb ischemia-reperfusion model (*in vivo*): evaluated by gene expression and histopathologic analyses	Suppressing the inflammatory response in mouse hindlimb post the tourniquet-induced ischemia-reperfusion; Reducing tissue edema, inflammatory exudate formation, and leukocyte infiltration; Decreasing the expression of VCAM-1, and E-selectin in the hindlimb tissue post ischemia-reperfusion.	(20)
Mouse COPD model (*in vivo*): porcine pancreatic elastase and lipopolysaccharide induced emphysema-exacerbation model	Attenuating inflammation in the short-term emphysema-exacerbation COPD mouse model; Reducing lung edema and permeability; Decreasing leukocyte infiltration, inflammatory cytokine expression, mucin production, and protease (MMP9 and MMP12) expression in the lung.	(29)

Based on the effects of GPR4 antagonists in other disease models, inhibition of GPR4 can be explored as a novel approach to mitigate COVID-19 complications. GPR4 antagonists potentially target several key aspects of COVID-19 pathophysiology (Figure 1). *First*, GPR4 antagonists may inhibit inflammatory responses and leukocyte infiltration in the lung and other affected organs of COVID-19 patients. Hyper-inflammatory responses and massive leukocyte infiltration are observed in COVID-19 patients exhibiting severe disease symptoms (4, 6). Inhibition of GPR4 can suppress the expression of inflammatory adhesion molecules, chemokines, and cytokines in vascular endothelial cells and subsequently decrease leukocyte-endothelium adhesion, extravasation and inflammatory responses (11–20, 29). *Second*, GPR4 antagonists may reduce vascular leakage, tissue edema and inflammatory exudate formation in COVID-19. Increased vascular permeability and disruption of epithelial and endothelial barriers in COVID-19 patients result in fluid accumulation and exudate formation in the lung, with impaired gas exchange and hypoxemia (4, 6). As shown in the hindlimb ischemia-reperfusion, arthritis, and COPD animal models, inhibition of GPR4 can reduce vessel permeability and tissue edema (17, 18, 20, 29). *Third*, GPR4 antagonists may attenuate vascular thromboembolism in COVID-19. Due to coagulopathy, endothelial dysfunction and hyper-inflammatory responses, vascular thromboembolism is a common complication in severely ill COVID-19 patients (31, 32). Activation of GPR4 increases endothelial cell adhesiveness and blood cell-endothelium interactions (11, 12, 16). Inhibition of GPR4 may lessen inflammatory response, blood cell-endothelium adhesion and aggregation, and thromboembolism (43). *Fourth*, GPR4 antagonists may decrease angiogenesis in COVID-19. While the pathophysiological significance is still unclear, angiogenesis is increased in the lung of COVID-19 patients (32). Inhibition of GPR4 hinders blood vessel formation by modulating the VEGF pathway (17, 18, 56). GPR4 antagonists can potentially curtail angiogenesis in COVID-19. *Fifth*, GPR4 antagonists may alleviate pain associated with COVID-19. Muscle aches, sore throat, headache, and chest pain are common symptoms of COVID-19. GPR4 is expressed in nociceptors such as dorsal root ganglion neurons and consequently aggravates inflammatory pain (17, 18, 57). Inhibition of GPR4 can potentially mitigate inflammatory pain in COVID-19 patients.

## Discussion

SARS-CoV-2 infection in the lung and other organs cause cellular injury and inflammatory responses in COVID-19 patients (4, 6, 10). Clinical manifestations of COVID-19 range widely from asymptomatic carriers to severe disease and death. Based on the current incomplete understanding of COVID-19 pathophysiology, therapeutic strategies have been directed toward anti-viral, anti-inflammatory, and anti-coagulatory agents. The applications of remdesivir, convalescent plasma, dexamethasone, tocilizumab, and low molecular weight heparin have achieved limited success in severely ill COVID-19 patients (4, 6). Because various factors are involved in COVID-19 pathophysiology, combination therapy targeting both the SARS-CoV-2 virus and the host inflammatory response may be required to achieve optimal treatment outcomes. A better understanding of COVID-19 pathophysiology will help develop novel therapeutic approaches.

We hypothesize that GPR4 plays an integral role in COVID-19 pathophysiology and inhibition of GPR4 can be explored as a novel approach to mitigate COVID-19 complications. G protein-coupled receptors (GPCRs) are the largest family of cell surface receptors that serve as pharmacological targets of ~34% of all FDA approved drugs (58). GPR4 antagonists have recently been developed and characterized. Consistent with its pro-inflammatory function, GPR4 inhibition by its antagonists alleviates inflammation, edema, and pain in preclinical disease models (15, 17, 18, 20, 29). To evaluate the potential therapeutic effects of GPR4 antagonists in COVID-19, the inhibitors can first be tested in preclinical animal models predisposed to infection with SARS-CoV-2, such as the human angiotensin-converting enzyme 2 (hACE2) transgenic mice and hamsters (59, 60). In addition to inflammatory responses, the effects of GPR4 antagonists on other COVID-19 complications such as blood vessel permeability, lung edema, vascular thromboembolism, and pain can also be evaluated in these preclinical animal models.

With regard to the safety profile and adverse effects of the GPR4 antagonists, an oral dose of 30–100 mg/kg (b.i.d.) is well tolerated in preclinical animal models without overt adverse effects (15, 17, 18, 20, 23, 29). The optimized GPR4 antagonist 13 (NE 52-QQ57) has no documented serious adverse effects on the cardiovascular and respiratory systems in mouse and rat models (23). Specifically, the GPR4 antagonist 13 (NE 52-QQ57) is selective for the GPR4 receptor and has no or minimal effects on other proton-sensing GPCRs or the common off-targets such as the H3 receptor and hERG channel (17). GPR4 antagonist 13 (NE 52-QQ57) does not affect hemodynamics, blood oxygen level dependent responses, or cerebral blood flow in rodents (23). It causes a slight reduction in the ventilatory response to 5 and 10% CO_2_ in non-anesthetized but not in anesthetized mice and rats (23). Moreover, phenotypic observations from GPR4 knockout mice indicate several facets of GPR4 functions. A small percentage of GPR4-null mice exhibit perinatal complications (61). Upon acid overload, GPR4-null mice have slightly decreased renal acid excretion (26). GPR4 is also involved in carbon dioxide chemosensing (62). Deletion of GPR4 is associated with lower blood pressure, lower binding to angiotensin II receptor, and increased insulin sensitivity (63, 64); these aspects are of particular interest as hypertension and diabetes are risk factors associated with COVID-19 mortality (4). The functional characteristics from knockout studies should be closely monitored when GPR4 antagonists are applied *in vivo*, although the biological effects from genetic knockout are not necessarily identical to pharmacological inhibition. Overall, the GPR4 antagonists exhibit a good pharmacological profile and oral bioavailability in preclinical animal models, providing a foundation for therapeutic evaluation in COVID-19 disease models.

Due to the complex pathophysiology of COVID-19, combination therapy is likely needed to achieve optimal treatment outcomes in COVID-19 patients with severe disease. In this respect, there are several strategies to apply GPR4 antagonists in combination with other therapeutic agents. One strategy is to combine GPR4 antagonists with anti-viral agents such as remdesivir to target both SARS-CoV-2 replication and the host hyper-inflammatory responses. Another strategy is to combine GPR4 antagonists with other anti-inflammatory agents such as dexamethasone, of which GPR4 antagonists target the endothelium-leukocyte interactions and dexamethasone targets immune cells. These strategies can be assessed in preclinical COVID-19 animal models and eventually patients. In summary, our central hypothesis is that GPR4 is a pro-inflammatory receptor involved in COVID-19 pathophysiology and GPR4 antagonists, whether as a single therapeutic agent or in combination with other agents, can be explored as a potential therapeutic approach to mitigate COVID-19 complications and may also find applications in other related diseases.

## Data Availability Statement

The original contributions presented in the study are included in the article/Supplementary Materials, further inquiries can be directed to the corresponding author/s.

## Author Contributions

LY conceived the project and drafted the manuscript. KO, MT, MM, SN, and JM contributed to valuable intellectual discussions and manuscript revision. All authors contributed to the article and approved the submitted version.

## Conflict of Interest

The authors declare that the research was conducted in the absence of any commercial or financial relationships that could be construed as a potential conflict of interest.

## Abbreviations

ACE2: angiotensin converting enzyme 2
ARDS: acute respiratory distress syndrome
ATF3: activating transcription factor 3
CCL2 (MCP-1): C-C motif chemokine ligand 2 (monocyte chemoattractant protein-1)
CCL3 (MIP-1α): C-C motif chemokine ligand 3 (macrophage inflammatory protein-1α)
CHOP: C/EBP homologous protein
COPD: chronic obstructive pulmonary disease
COVID-19: coronavirus disease 19
COX2: cyclooxygenase-2
CXCL10 (IP-10): C-X-C motif chemokine ligand 10 (interferon gamma-induced protein 10)
DSS: dextran sulfate sodium
ER: endoplasmic reticulum
FDA: Food and Drug Administration
GPCR: G protein-coupled receptor
GPR4: G protein-coupled receptor 4
IBD: inflammatory bowel disease
ICAM-1: Intercellular Adhesion Molecule 1
ICU: intensive care unit
IgG: immunoglobulin G
IL: interleukin
iNOS: inducible nitric oxide synthase
KO: knockout
MMP: matrix metalloproteinase
NF-κB: nuclear factor kappa-light-chain-enhancer of activated B cells
PGE2: prostaglandin E2
pH: potential of hydrogen
RAAS: renin-angiotensin-aldosterone system
SARS-CoV-2: severe acute respiratory syndrome coronavirus 2
TNF-α: tumor necrosis factor-α
VCAM-1: vascular cell adhesion molecule 1
VEGF: vascular endothelial growth factor.
